# Power Ultrasound- and Organic Acid-Based Hurdle Technology to Reduce *Listeria monocytogenes* and *Salmonella enterica* on Whole Apples and Peaches

**DOI:** 10.3390/foods14101744

**Published:** 2025-05-14

**Authors:** Bashayer A. Khouja, Hina Mathias, Mayura Joshi, Megan L. Fay, Supriya Korade, Catherine W. Y. Wong, Diana S. Stewart, Xinyi Zhou, Wei Zhang, Joelle K. Salazar

**Affiliations:** 1 Division of Food Processing Science and Technology, U. S. Food and Drug Administration, Bedford Park, IL 60501, USA; 2 Illinois Institute of Technology, Department of Food Science and Nutrition, Bedford Park, IL 60501, USA

**Keywords:** antimicrobials, fruit, *Listeria*, power ultrasound, *Salmonella*

## Abstract

Fresh produce, such as peaches and apples, are agricultural commodities, making them susceptible to contamination by foodborne pathogens such as *Listeria monocytogenes* and *Salmonella enterica*. Traditional methods, such as chlorine washes, have limitations related to antimicrobial efficacy, prompting interest in alternative techniques, such as power ultrasound. This study evaluated the use of power ultrasound, alone and combined with organic acids (citric, lactic, and malic), to reduce pathogen populations on whole apples and peaches. Pathogen cocktails of *L. monocytogenes* and *S. enterica* were spot-inoculated on fruit surfaces at an initial population level of 8–9 log CFU/fruit. The fruits were then submerged in water or citric, malic, or lactic acid at concentrations of 1%, 2%, or 5% alone or with power ultrasound treatment at 40 kHz for 2, 5, or 10 min. Results revealed that treatment conditions on apples exhibited significantly greater pathogen reduction than on peaches, likely due to the smoother surface topology on apples compared to the rougher, trichome-covered peach surfaces. Between the two pathogens, *L. monocytogenes* exhibited significantly greater resistance to treatments, resulting in maximum reductions of approximately 4 log CFU/fruit. In contrast, treatments were more effective against *S. enterica,* as lactic acid alone reduced *S. enterica* populations by >6 log CFU/fruit. Malic acid was the second-most effective organic acid against *S. enterica, leading to >4 log CFU/fruit reduction.* Synergistic antimicrobial effects were observed when organic acids were used in combination with power ultrasound. For instance, an additional reduction of 2–3 log CFU/fruit was achieved for *S. enterica* compared to the use of organic acid treatments alone. These findings support the use of organic acid and power ultrasound in hurdle as an effective strategy to mitigate foodborne pathogen risks on whole fruits such as apples and peaches. Further research would be helpful to optimize and validate such hurdle treatments for inactivating a broader spectrum of microbial pathogens on diverse produce surfaces.

## 1. Introduction

Foodborne pathogens, such as *Salmonella enterica* (*S. enterica*) and *Listeria monocytogenes* (*L. monocytogenes*), pose significant risks to food safety and public health, particularly in the fresh produce sector [1,2]. Contamination with foodborne pathogens can occur at any stage of production, from pre-harvest to post-harvest handling, leading to outbreaks of foodborne illnesses and highlighting the need for effective pathogen reduction strategies. Fresh fruits, such as apples and peaches, which are often eaten raw, are of particular concern for foodborne infections, making the development of more effective and consumer-safe pathogen reduction methods critical [1].

In the last decade, there have been various recalls and several notable outbreaks of *L. monocytogenes* and *S. enterica* linked to contaminated whole apples and peaches, underscoring the need for improved safety measures [3,4,5,6,7,8,9,10,11]. In 2014 and 2017, two multi-state listeriosis outbreaks occurred in the U.S., which were traced back to prepackaged caramel apples [5,10]. The first outbreak, in 2014, resulted in 35 cases, 34 hospitalizations, and seven deaths. This outbreak highlighted the risk of contamination not only in fresh whole apples but also in processed apple products (i.e., caramel-coated apples), where pathogens can thrive in niches created during processing [5]. In 2017, caramel apples were again the implicated outbreak vehicle, resulting in three cases, all of whom were hospitalized [10].

Similarly, fresh whole peaches have also been associated with foodborne illness outbreaks [3,6,11]. In 2020, an outbreak of salmonellosis in the U.S. and Canada was linked to peaches, resulting in 101 cases and 28 hospitalizations across 17 states [3]. This outbreak represented the first case of *S. enterica* contamination linked to peaches. In 2023, a significant listeriosis outbreak was linked to stone fruits, which included peaches, as well as nectarines and plums [6]. This multi-state outbreak resulted in 11 illnesses, 10 hospitalizations, and one death. Taken together, these outbreaks highlight the ongoing challenges of controlling *L. monocytogenes* and *S. enterica* contamination in fresh apples and peaches.

Traditional methods for reducing pathogens on fresh produce based on chlorine water washes have many limitations, including potentially hazardous chemical residues, limited antimicrobial efficacy, and growing consumer concerns regarding long-term environmental impacts and sustainability [12,13]. Thus, there is an increasing demand in developing alternative and organic techniques that are both effective and safe for consumers. Among these methods is power ultrasound technology, which uses frequencies between 20 and 100 kHz and is routinely employed in the medical field [14]. Power ultrasound technology has gained increased attention in food safety applications due to its ability to efficiently disrupt bacterial cells through cavitation, which can enhance the penetration and action of antimicrobial agents [15].

Studies have demonstrated that power ultrasound, either used alone or in combination with antimicrobials including chlorine, chlorine dioxide, peracetic acid, or other organic acids, is capable of reducing the population of foodborne pathogens and native microbiota on fresh produce, including cabbage, lettuce, spinach, and tomatoes. Pathogens are reduced by <1–6 log CFU depending on the produce matrix, treatment conditions, and the treatment length [16,17,18,19,20,21,22,23]. However, no information is available in the published literature on the effectiveness of organic acids coupled with power ultrasound treatment for the reduction of foodborne pathogens, like *L. monocytogenes* and *S. enterica*, on whole fruits such as apples and peaches.

The combination of power ultrasound and organic acids presents a promising synergistic approach for pathogen reduction on fresh produce. Organic acids, such as acetic, lactic, and malic acid, are generally recognized safe (GRAS) and have been shown to exhibit antimicrobial properties [24]. When used in conjunction with power ultrasound, these acids can potentially achieve higher microbial reduction by disrupting the cell membrane of pathogens more effectively [22]. The aim of this study was to evaluate the efficacy of power ultrasound alone or when coupled with organic acid treatment to reduce *L. monocytogenes* and *S. enterica* on fresh whole apples and peaches.

## 2. Materials and Methods

### 2.1. Strains and Culture Conditions

A four-strain cocktail of *L. monocytogenes* and a four-strain cocktail of *S. enterica* were used in this study. For *L. monocytogenes*, the strains used were ScottA (clinical isolate), LS3132 (avocado isolate), LS810 (cantaloupe isolate), and 573-035 (caramel apple outbreak isolate). For *S. enterica*, the strains used were Enteritidis PT30 (ATCC BAA-1045, almond isolate), Agona (447967, roasted oats cereal isolate), Alachua (CFSAN107331, peach leaf isolate), and Poona 8785 (CFSAN038692, cucumber isolate). All strains were rifampicin-resistant (100 µg/mL). Strains were cultured individually in 25 mL of tryptic soy broth (TSB; Becton, Dickinson and Company, Sparks, MD, USA) and incubated at 37 °C for 16–18 h. The individual cultures were pelleted down by centrifugation for 5 min at 6000 rpm and cell pellets were washed once with 10 mL Butterfield’s phosphate buffer (BPB, pH 7.4). The pelleted cells were each resuspended in 2.5 mL BPB and combined (10 mL total) to achieve an initial concentration of approximately 10 log CFU/mL. The initial population levels of the *L. monocytogenes* and *S. enterica* cocktails were verified by serially diluting and plating onto brain heart infusion agar (BHIA; Becton, Dickinson and Co.).

### 2.2. Preparation of Washing Treatments

#### 2.2.1. Organic Acids

Citric, lactic, and malic acids (Fisher Scientific, Fair Lawn, NJ, USA) were used in this study. Organic acids were prepared at concentrations of 1%, 2%, or 5% *w*/*v* (for citric and malic acid) or *v*/*v* (for lactic acid) in sterile water. The pH values of the organic acid solutions were measured in triplicate using a pH meter (Mettler Toledo, Columbus, OH, USA) (Table 1). Freshly made solutions were utilized for each of three independent trials.

#### 2.2.2. Power Ultrasound

Ten liter-capacity power ultrasound bath units were used in this study (TH-SPQXJ-40A, Vevor, Shanghai, China) at 40 kHz. The units were degassed for 10 min prior to use. Prior to the experiments in this study, a preliminary study was conducted to evaluate the differences, if any, between *L. monocytogenes* or *S. enterica* population reductions on apples or peaches when power ultrasound treatment occurred using 2 L-capacity glass beakers or 710 mL-capacity plastic stomacher bags [25]. No statistical differences were observed in the reductions of either pathogen on either produce type, and thus the treatment experiments in this study utilized plastic stomacher bags.

### 2.3. Produce Preparation and Inoculation

Fresh whole Gala apples (*Malus domestica* var Gala) and yellow flesh peaches (*Prunus persica* var Red Haven; with intact trichome) weighing an average of 108.38 ± 6.59 and 149.94 ± 66.73 g, respectively, were purchased from local retail grocers and stored at ambient temperature for up to 24 h prior to experiments. Any apples or peaches with bruises or other visual defects were discarded. Apples and peaches were placed stem-side up onto foil trays within a biosafety cabinet. Surface inoculation of the apples and peaches was conducted using 100 µL of either the *L. monocytogenes* or *S. enterica* cocktail: approximately 7–8 spots were pipetted onto the surface and the stem end of each fruit. The inoculum was allowed to dry on the apples and peaches in the biosafety cabinet with the blower on for 1 h.

### 2.4. Treatment of Produce

Following the 1 h drying period, apples and peaches were submerged in 225 mL of water or citric, malic, or lactic acid at 1%, 2%, or 5% alone or with power ultrasound treatment. Treatment times were 2, 5, or 10 min. All treatments were conducted at room temperature (20–22 °C). During ultrasound treatment, water temperatures were increased approximately 0.5 °C/min, resulting in temperatures of 21–23, 22.5–24.5, and 25–27 °C after 2, 5, and 10 min, respectively.

Immediately following the treatment, apples and peaches were removed from the treatment solution and submerged in 225 mL of BPB. For each trial, triplicate samples were evaluated for each produce type, pathogen, and treatment combination. Three independent trials were conducted.

### 2.5. Enumeration of L. monocytogenes and S. enterica

Apples and peaches were stomached for 1 min (JumboMix 3500 W CC Lab Blender, Interscience, Woburn, MA, USA). Samples were then serially diluted and plated onto brain heart infusion agar supplemented with rifampicin (BHIA^rif^) (100 µg/mL). Agar plates were incubated at 37 °C for 24–48 h prior to enumeration. Pathogen population data are expressed as log CFU/fruit.

### 2.6. Statistical Analysis

Three independent trials were performed with triplicate samples for each condition (n = 9). Significant differences in population reductions of *L. monocytogenes* and *S. enterica* on apples or peaches treated with water for 2, 5, or 10 min without or with power ultrasound were statistically determined using one-way analysis of variance (ANOVA) with Tukey’s post hoc test. Significant differences in population reductions of *L. monocytogenes* or *S. enterica* on apples or peaches treated with citric, malic, or lactic acid at 1%, 2%, or 5% for 2, 5, or 10 min without or with power ultrasound were also determined using ANOVA with Tukey’s post hoc test. A *p*-value ≤ 0.05 was considered significant.

## 3. Results

### 3.1. Efficacy of Water Alone or in Combination with Power Ultrasound to Reduce Pathogen Populations on Fruit

**Figure 1:**
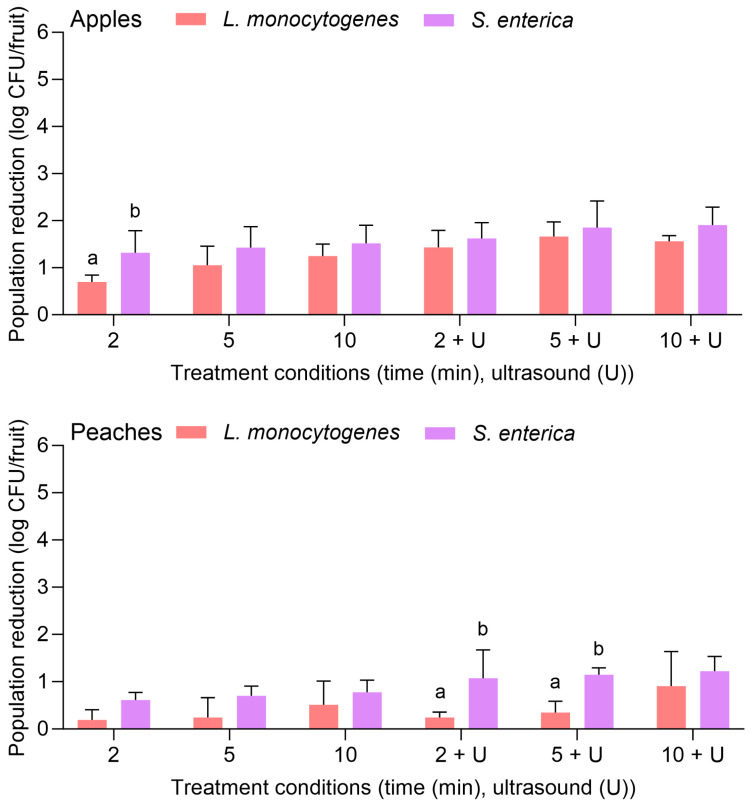
Population reductions of *Listeria monocytogenes* and *Salmonella enterica* on apples and peaches treated with water alone for 2, 5, or 10 min with or without power ultrasound (U). Data are mean values ± standard deviation (n = 9). Different lowercase letters indicate that *L. monocytogenes* and *S. enterica* population reductions are significantly different in the same treatment groups (the same treatment length without or with ultrasound) on the same fruit. Data within a treatment group on the same fruit without lowercase letters are not significantly different.

Figure 1 depicts the population reductions of both *L. monocytogenes* and *S. enterica* on apples and peaches treated with water for 2, 5, or 10 min with or without power ultrasound. For apples, the initial inoculation levels were 8.92 ± 0.42 and 8.17 ± 0.37 log CFU/fruit for *S. enterica* and *L. monocytogenes*, respectively. With water alone, *L. monocytogenes* was reduced by 0.70 ± 0.15 and 1.24 ± 0.26 log CFU/fruit after 2 and 10 min, respectively. The combination of water with the power ultrasound treatment significantly increased the pathogen reduction for the same treatment lengths: populations were reduced by 1.43 ± 0.26 and 1.56 ± 0.12 log CFU/fruit after 2 and 10 min, respectively. No significant difference was observed between the reductions of *L. monocytogenes* and *S. enterica* on apples when the same treatments and treatment lengths were used, with the exception of the 2 min water wash, where *S. enterica* was reduced by 1.32 ± 0.47 log CFU/fruit.

For peaches, the initial inoculation levels were 8.54 ± 0.68 and 8.67 ± 0.41 log CFU/fruit for *S. enterica* and *L. monocytogenes*, respectively. Population reductions were markedly lower on peaches for both pathogens. For *L. monocytogenes*, reductions were <1 log CFU/fruit for all tested treatment lengths, regardless of the use of power ultrasound. The population was reduced by 0.91 ± 0.73 log CFU/fruit after the 10 min water treatment with power ultrasound. For *S. enterica*, population reductions on peaches were also <1 log CFU/fruit for all treatment lengths when water was used alone. In combination with power ultrasound, population reductions were >1 log CFU/fruit, with the greatest reduction observed after 10 min (1.22 ± 0.21 log CFU/fruit). Compared to *L. monocytogenes*, *S. enterica* was more significantly reduced on peaches when treated with water for 2 or 5 min with the combination of power ultrasound.

### 3.2. Reduction in Pathogen Populations on Apples Treated with Organic Acids Alone or in Combination with Power Ultrasound

**Figure 2:**
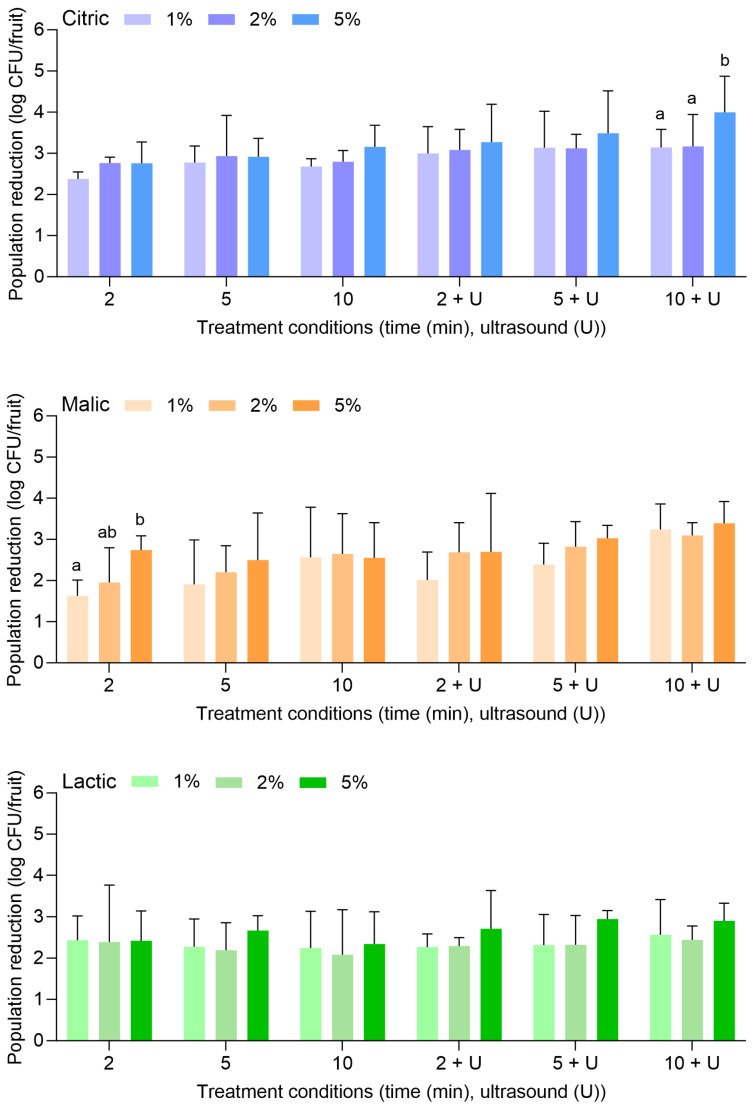
Population reductions of *Listeria monocytogenes* on apples treated with citric, malic, or lactic acid at 1%, 2%, or 5% for 2, 5, or 10 min with or without power ultrasound (U). Data are mean values ± standard deviation (n = 9). Different lowercase letters indicate that means are significantly different in the same treatment groups (the same treatment length without or with ultrasound). Data within a treatment group without lowercase letters are not significantly different.

Figure 2 displays the population reductions of *L. monocytogenes* on apples treated with citric, malic, or lactic acid at 1%, 2%, or 5% for 2, 5, or 10 min alone or in combination with power ultrasound. With citric acid alone, *L. monocytogenes* populations were reduced by 2.38 ± 0.17 (1%, 2 min) to 3.16 ± 0.52 log CFU/fruit (5%, 10 min). With the combination of citric acid and power ultrasound, populations were reduced by 3.00 ± 0.65 (1%, 2 min) to 4.00 ± 0.88 log CFU/fruit (5%, 10 min). Compared to water alone or in combination with power ultrasound (Figure 1), populations of *L. monocytogenes* were further reduced by approximately 1–2 log CFU/fruit with citric acid.

Malic and lactic acid were less effective than citric acid for reducing *L. monocytogenes* on apples. With malic acid alone, *L. monocytogenes* populations were reduced by 1.63 ± 0.38 (1%, 2 min) to 2.55 ± 0.86 log CFU/fruit (5%, 10 min). With the combination of malic acid and power ultrasound, populations were reduced by 2.02 ± 0.68 (1%, 2 min) to 3.39 ± 0.53 log CFU/fruit (5%, 10 min). With lactic acid alone, populations were reduced by 2.43 ± 0.59 (1%, 2 min) to 2.34 ± 0.78 log CFU/fruit (5%, 10 min). With the combination of lactic acid and power ultrasound, populations were reduced by 2.27 ± 0.32 (1%, 2 min) to 2.90 ± 0.42 log CFU/fruit (5%, 10 min). In general, the increase in the acid concentrations, the treatment lengths, or the use of ultrasound did not result in significantly greater reductions of *L. monocytogenes* on apples.

**Figure 3:**
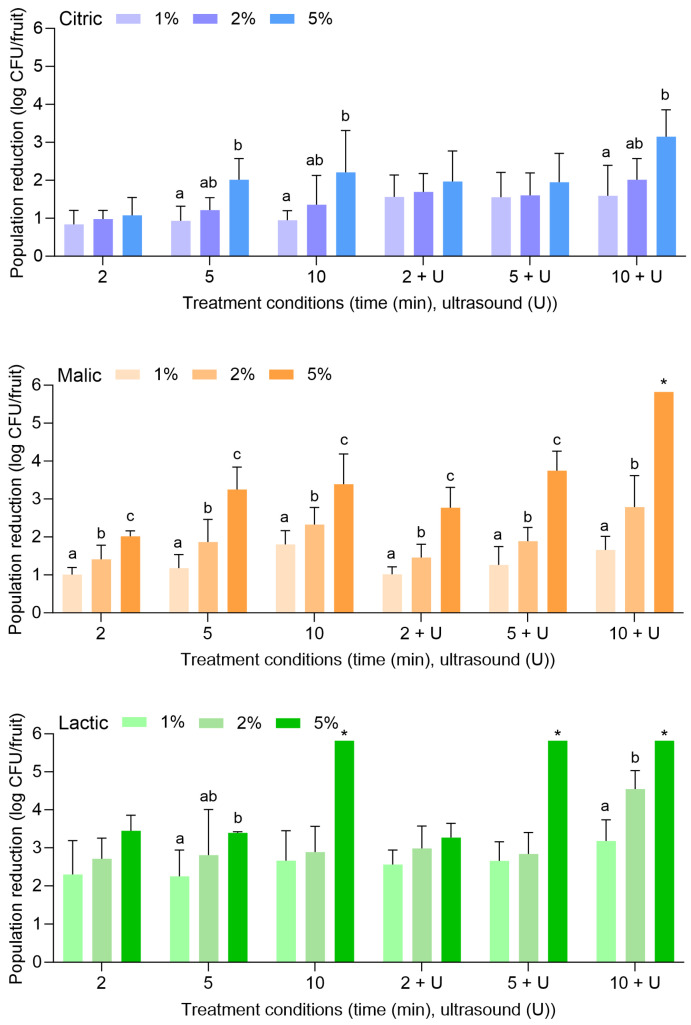
Population reductions of *Salmonella enterica* on apples treated with citric, malic, or lactic acid at 1%, 2%, or 5% for 2, 5, or 10 min with or without power ultrasound (U). Data are mean values ± standard deviation (n = 9). Different lowercase letters indicate that means are significantly different in the same treatment groups (the same treatment length without or with ultrasound). Data within a treatment group without lowercase letters are not significantly different. The asterisk (*) indicates that population reductions were >5.82 log CFU/unit.

Figure 3 displays the population reductions of *S. enterica* on apples treated with citric, malic, or lactic acid at 1%, 2%, or 5% for 2, 5, or 10 min alone or in combination with power ultrasound. With citric acid alone, *S. enterica* populations were reduced by 0.84 ± 0.37 (1%, 2 min) to 2.21 ± 1.11 log CFU/fruit (5%, 10 min). With the combination of citric acid and power ultrasound, populations were reduced by 1.56 ± 0.65 (1%, 2 min) to 3.15 ± 0.71 log CFU/fruit (5%, 10 min). It is noted that reductions of *L. monocytogenes* on apples treated with the same combinations of citric acid concentrations and treatment lengths were more significant than *S. enterica*.

Malic and lactic acid were substantially more effective than citric acid for the reduction of *S. enterica* on apples, especially when the higher concentration (i.e., 5%) was used. With malic acid alone, *S. enterica* populations were reduced by 1.00 ± 0.19 (1%, 2 min) to 3.39 ± 0.80 log CFU/fruit (5%, 10 min). With the combination of malic acid and power ultrasound, populations were reduced by 1.02 ± 0.20 (1%, 2 min) to >5.82 log CFU/fruit (5%, 10 min). With lactic acid alone, populations were reduced by 2.30 ± 0.88 (1%, 2 min) to >5.82 log CFU/fruit (5%, 10 min). With the combination of lactic acid and power ultrasound, populations were reduced by 2.66 ± 0.50 (1%, 2 min) to >5.82 log CFU/fruit (5%, 10 min). *S. enterica* population reductions of >5.82 log CFU/fruit were achieved with 5% malic acid for 10 min with power ultrasound, for 5% lactic acid for 10 min without power ultrasound, and for 5% lactic acid for 5 and 10 min with power ultrasound.

### 3.3. Reduction of Pathogen Populations on Peaches Treated with Organic Acids Alone or in Combination with Power Ultrasound

**Figure 4:**
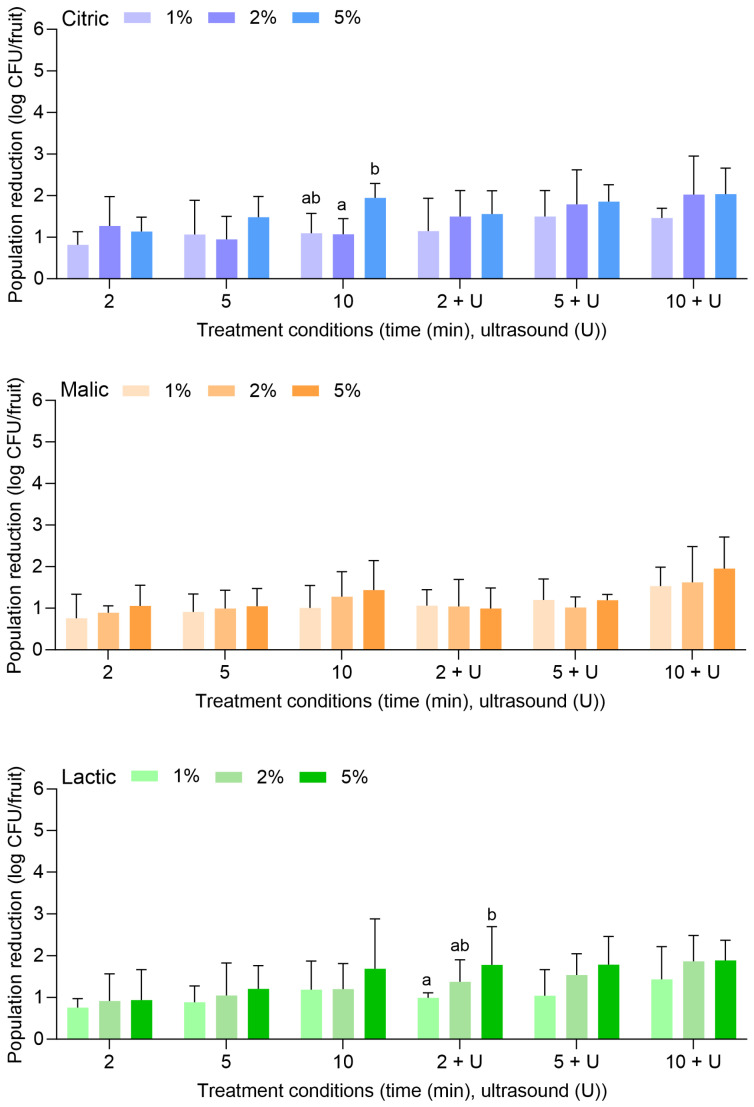
Population reductions of *Listeria monocytogenes* on peaches treated with citric, malic, or lactic acid at 1%, 2%, or 5% for 2, 5, or 10 min with or without power ultrasound (U). Data are mean values ± standard deviation (n = 9). Different lowercase letters indicate that means are significantly different in the same treatment groups (the same treatment length with or without ultrasound). Data within a treatment group without lowercase letters are not significantly different.

Figure 4 displays the population reductions of *L. monocytogenes* on peaches treated with citric, malic, or lactic acid at 1%, 2%, or 5% for 2, 5, or 10 min alone or in combination with power ultrasound. In general, treatments were less effective for the reduction of *L. monocytogenes* on peaches than that observed for apples. With citric acid alone, *L. monocytogenes* populations were reduced by 0.88 ± 0.17 (1%, 2 min) to 1.95 ± 0.34 log CFU/fruit (5%, 10 min). With the combination of citric acid and power ultrasound, populations were reduced by 1.15 ± 0.79 (1%, 2 min) to 2.04 ± 0.62 log CFU/fruit (5%, 10 min). Compared to water alone or in combination with power ultrasound (Figure 1), populations of *L. monocytogenes* were only further reduced by >1 to 1 log CFU/fruit with the use of citric acid.

With malic acid alone, *L. monocytogenes* populations were reduced by 0.76 ± 0.58 (1%, 2 min) to 1.44 ± 0.71 log CFU/fruit (5%, 10 min). With the combination of malic acid and power ultrasound, populations were reduced by 1.06 ± 0.39 (1%, 2 min) to 1.95 ± 0.76 log CFU/fruit (5%, 10 min). With lactic acid alone, populations were reduced by 0.76 ± 0.21 (1%, 2 min) to 1.69 ± 1.19 log CFU/fruit (5%, 10 min). With the combination of lactic acid and power ultrasound, populations were reduced by 0.99 ± 0.12 (1%, 2 min) to 1.89 ± 0.48 log CFU/fruit (5%, 10 min). Overall, the increase in the acid concentrations, the treatment lengths, or the use of ultrasound did not result in significantly greater reductions of *L. monocytogenes* on peaches.

**Figure 5:**
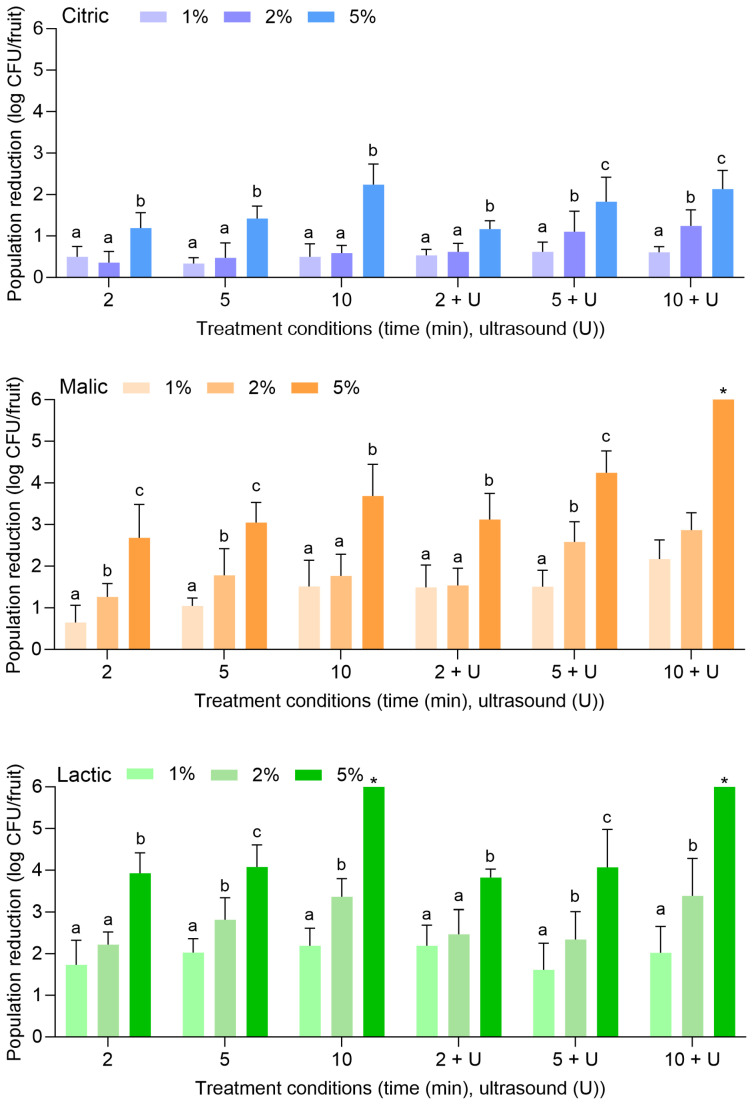
Population reductions of *Salmonella enterica* on peaches treated with citric, malic, or lactic acid at 1%, 2%, or 5% for 2, 5, or 10 min with or without power ultrasound (U). Data are mean values ± standard deviation (n = 9). Different lowercase letters indicate that means are significantly different in the same treatment groups (the same treatment length with or without ultrasound). Data within a treatment group without lowercase letters are not significantly different. The asterisk (*) indicates that population reductions were >6.32 log CFU/unit.

Figure 5 displays the population reductions of *S. enterica* on peaches treated with citric, malic, or lactic acid at 1%, 2%, or 5% for 2, 5, or 10 min alone or in combination with power ultrasound. With citric acid alone, *S. enterica* populations were reduced by 0.50 ± 0.25 (1%, 2 min) to 2.24 ± 0.50 log CFU/fruit (5%, 10 min). With the combination of citric acid and power ultrasound, populations were reduced by 0.53 ± 0.15 (1%, 2 min) to 2.13 ± 0.45 log CFU/fruit (5%, 10 min). The use of 1% or 2% citric acid did not result in further reductions compared to when water was used alone or with power ultrasound (Figure 1). The use of 5% citric acid was more effective at all treatment lengths, further reducing *S. enterica* on peaches when treatment occurred alone or when power ultrasound was used.

Similarly to what was observed for apples, malic and lactic acid were also more substantially effective than citric acid for the reduction of *S. enterica* on peaches, especially when the higher concentration (i.e., 5%) was used. With malic acid alone, *S. enterica* populations were reduced by 0.65 ± 0.41 (1%, 2 min) to 3.69 ± 0.76 log CFU/fruit (5%, 10 min). With the combination of malic acid and power ultrasound, populations were reduced by 1.49 ± 0.54 (1%, 2 min) to >6.32 log CFU/fruit (5%, 10 min). With lactic acid alone, populations were reduced by 1.73 ± 0.59 (1%, 2 min) to >6.32 log CFU/fruit (5%, 10 min). With the combination of lactic acid and power ultrasound, populations were reduced by 2.19 ± 0.49 (1%, 2 min) to >6.32 log CFU/fruit (5%, 10 min). *S. enterica* population reductions of >6.32 log CFU/fruit were achieved with 5% malic acid for 10 min with power ultrasound, for 5% lactic acid for 10 min without power ultrasound, and for 5% lactic acid for 5 and 10 min with power ultrasound.

## 4. Discussion

With the demand for and consumption of fresh produce increasing in recent years, the number of foodborne outbreaks associated with fresh produce has also increased [26,27]. Fresh produce is a vector for foodborne bacterial pathogens, providing suitable environments for the survival, persistence, and even proliferation of these organisms. Fresh produce can become contaminated with pathogens at various pre-and post-harvesting stages [1]. To reduce the microbial burden, fresh produce is minimally processed, which often includes washing with water supplemented with antimicrobials. While chlorine and peroxyacetic acid are generally used to reduce the microbial population and to prevent possible pathogen cross-contamination in the wash water, these antimicrobials can produce volatile compounds and have been shown to be less effective with increased organic load, with certain produce items, and with select bacterial pathogens [28,29,30,31].

This study evaluated the use of select organic acids either alone or in combination with power ultrasound to reduce *L. monocytogenes* and *S. enterica* on two fresh fruits, apples and peaches. Marked differences were observed in the reduction of pathogens depending on the fruit surface, the type of organic acid used, and the use of the combined organic acid and power ultrasound treatment. Apples and peaches were selected for this study due to their association with foodborne outbreaks and due to their very different surface characteristics. Results of this study indicate that pathogen populations on apples, especially *S. enterica*, were more susceptible to the combined treatment of organic acids and power ultrasound than those on peaches. While the apples used in this study had a smooth surface topology, the peaches had a rougher surface with intact trichomes, possibly aiding bacterial attachment, attachment strength, or allowing the bacteria to hide in crevices which may have limited the effectiveness of the treatments.

Studies have shown that produce surface topology, including roughness, can impact bacterial attachment, removal, and the effectiveness of sanitizers [16,28,32,33]. One study determined that *S. enterica* more preferentially attached to the stem end, calyx, and the injured surfaces of apples than to the smoother uninjured surfaces [32]. Sanitizers, including hydrogen peroxide, trisodium phosphate, calcium hypochlorite, and sodium hypochlorite, were not as effective against *S. enterica* on the rougher apple surfaces. In another study, the combination of sodium hypochlorite or peroxyacetic acid with power ultrasound was more effective at reducing *L. monocytogenes* and *S. enterica* on the smooth surface of grape tomatoes than on the rougher surfaces of spinach and iceberg lettuce [16]. Power ultrasound technology has also been shown to be more effective at reducing pathogens on produce surfaces which are smoother [34].

This study also demonstrated varied sanitizing efficacy among different bacterial species. For instance, reduction of *L. monocytogenes* on the fruit surfaces was not as significant with the organic acid and power ultrasound treatments as *S. enterica*. Specifically, greater reductions of *S. enterica*, (>5.82 and >6.32 log CFU/fruit on apples and peaches, respectively) were achieved with the combined organic acid and power ultrasound hurdle technology. For *L. monocytogenes*, the greatest reductions were 4.00 ± 0.88 log CFU/fruit on apples and only 2.04 ± 0.62 log CFU/fruit on peaches. Studies have determined that Gram-positive bacteria (e.g., *L. monocytogenes*) are more resistant than Gram-negative bacteria (e.g., *S. enterica*) to stressors, including power ultrasound [35,36]. It is thought the more tightly adherent layer of peptidoglycan in Gram-positive bacteria may contribute to this resistance. Power ultrasound creates damage to the cell walls and cell structures of bacteria and more so for Gram-negative bacteria, thus resulting in greater detachment from produce surfaces and/or inactivation.

The greatest synergistic effect of the combination treatment of organic acid and power ultrasound was observed with *S. enterica* on both apples and peaches when malic and lactic acids were used at the higher concentration (i.e., 5%). For example, treatment of apples for 10 min with 5% malic acid alone reduced *S. enterica* by 3.39 ± 0.78 log CFU/fruit, whereas the pathogen was reduced by >5.82 log CFU/fruit with the combination of power ultrasound for the same treatment length (a further reduction of approximately 2.43 log CFU/fruit). Similarly, treatment of apples for 5 min with 5% lactic acid alone reduced *S. enterica* by 3.39 ± 0.03 log CFU/fruit, whereas the pathogen was reduced by >5.82 log CFU/fruit with the combination of power ultrasound for the same treatment length (also a further reduction of approximately 2.43 log CFU/fruit). When peaches were treated with 5% malic acid for 10 min, *S. enterica* was reduced by 3.69 ± 0.76 log CFU/fruit. With the incorporation of power ultrasound, the pathogen was reduced by >6.32 log CFU/fruit (a further reduction of approximately 2.63 log CFU/fruit).

The use of organic acid and power ultrasound hurdle technology has been previously shown to have synergistic effects for certain foodborne pathogens on select produce matrices [22,37]. For example, romaine lettuce treated with citric, lactic, or malic acid with the combination of power ultrasound resulted in an additional 0.8- to 1.0-log reduction of *E. coli* O157:H7, *S. enterica*, and *L. monocytogenes* compared to when the treatments were individually applied [22]. In another study, cherry tomatoes treated with lactic acid in combination with power ultrasound resulted in an additional 0.9-log reduction of *S. enterica* compared to individual treatments [37]. For radish, treatment with lactic acid and power ultrasound resulted in an additional 0.5- to 4.0-log reduction in *E. coli* and *L. monocytogenes* populations compared to when the treatments were individually applied.

This study evaluated the efficacy of organic acids, power ultrasound, and combined hurdle treatments to reduce *L. monocytogenes* and *S. enterica* on fresh whole apples and peaches. For even the longest treatment length evaluated in this study (10 min), no physical appearance or texture changes were observed for either apples or peaches. It is noted that power ultrasound treatment may result in changes to the fruit, including quality and sensory characteristics, color, or biochemical properties, especially if the length of treatment was increased. Results of this study suggest that produce matrix topology may also play a role in the effectiveness of the treatments, as greater pathogen reductions were achieved on apples than peaches. *S. enterica* appeared to be more sensitive to the organic acid and power ultrasound treatment, as indicated by greater log reductions on both apples and peaches. Synergistic effects of the hurdle technology were also observed, especially for *S. enterica* on both fruit matrices with the use of malic and lactic acids. Results suggest that the combination of organic acids and power ultrasound may be an effective hurdle technology to reduce foodborne pathogens from select fruit matrices. Additional future studies would be required to determine if the organic acids and power ultrasound hurdle technology can be effective against other foodborne pathogens on other types of fresh produce and if there is a synergistic effect with the combination of different organic acids together with and without power ultrasound to further reduce pathogenic populations across different fresh produce matrices and topology. Scaled-up studies with actual processing equipment, lower microbial load, and cost-effectiveness analysis would further help to validate this hurdle technology for practical industry use.

## Figures and Tables

**Table 1:** The pH of the organic acid treatments used in this study. Data are mean values ± standard deviation (n = 9).

Organic Acid	pH ± SD		
	1%	2%	5%
Citric	2.52 ± 0.22	2.41 ± 0.29	2.08 ± 0.20
Malic	2.19 ± 0.26	2.10 ± 0.35	1.79 ± 0.56
Lactic	2.20 ± 0.25	2.03 ± 0.32	1.76 ± 0.29

## Data Availability

The original contributions presented in this study are included in the article. Further inquiries can be directed to the corresponding author.
